# A Review of Non-^1^H RF Receive Arrays in Magnetic Resonance Imaging and Spectroscopy

**DOI:** 10.1109/OJEMB.2020.3030531

**Published:** 2020-10-13

**Authors:** Matthew Wilcox, Steven M. Wright, Mary McDougall

**Affiliations:** Texas A&M University 14736 College Station TX 77843 USA

**Keywords:** Array, magnetic resonance imaging, magnetic resonance spectroscopy, multi-nuclear, RF Coils

## Abstract

It is now common practice to use radiofrequency (RF) coils to increase the signal-to-noise ratio (SNR) in 1H magnetic resonance imaging and spectroscopy experiments. Use of array coils for non-1H experiments, however, has been historically more limited despite the fact that these nuclei suffer inherently lower sensitivity and could benefit greatly from an increased SNR. Recent advancements in receiver technology and increased support from scanner manufacturers have now opened greater options for the use of array coils for non-1H magnetic resonance experiments. This paper reviews the research in adopting array coil technology with an emphasis on studies of the most commonly studied non-1H nuclei including 31P, 13C, 23Na, and 19F. These nuclei offer complementary information to 1H imaging and spectroscopy and have proven themselves important in the study of numerous disease processes. While recent work with non-1H array coils has shown promising results, the technology is not yet widely utilized and should see substantial developments in the coming years.

## I. Introduction

Most people are familiar with magnetic resonance imaging (MRI) due to the ubiquity of the technique as a medical diagnostic and monitoring tool, particularly when imaging using the ^1^H nucleus. Magnetic resonance spectroscopy (MRS), though less utilized clinically than MRI, is a related technique potentially providing new layers of information. Despite arriving much later than spectroscopy techniques, MR imaging [1]–[3] has overshadowed MRS in clinical application for the last 30 years. Still, research in both ^1^H and non-^1^H MRS has actively proceeded due to the rich promise of non-invasive *in vivo* metabolic and chemical studies. Goal, methods, results, conclusion. To this end, array coils [4] have been developed and utilized for both non-^1^H spectroscopy and non-^1^H imaging applications.

Compared to detection of the ^1^H nucleus, the detection of most non-^1^H nuclei (often referred to as X-nuclei) is significantly more challenging. Though most NMR researchers are familiar with the relationship between SNR and field strength, here we are more concerned with the relationship between SNR and γ at a constant field strength. The maximum received NMR signal from a nucleus is roughly proportional to the cube of its gyromagnetic ratio γ [5]–[7]. This relationship is due to a combination of effects: the larger transition energy difference between the two nuclear spin states which leads to a greater Boltzmann population difference of these states, the higher magnetic moment of the spins, and the higher precession rate which leads to a greater induced signal in the detection coil through Faraday's Law. General relationships between γ and noise level are complicated by the fact that coils used for low- γ nuclei may be coil-noise dominated as opposed to the typically sample-noise dominated coils for ^1^H experiments. However, it has been said that the noise contribution is generally taken to increase with γ^1/2^, leading to an overall increase of signal-to-noise ratio (SNR) proportional to γ^5/2^ [5], [8], [9]. It should be noted that depending on the nature of the noise source (i.e., sample-noise dominance versus coil-noise dominance), other relationships between SNR and γ may be derived, but SNR is proportional to at least γ^2^ [6], [10]. Because all X-nuclei have a lower γ than that of the ^1^H nucleus, they suffer from comparatively decreased sensitivity.

Even assuming equivalent concentrations, low natural abundance for the NMR-active isotopes of some of these X-nuclei further decreases the achievable signal. The combination of natural abundance and the effects mentioned above is termed nuclear receptivity and represents the overall ease of acquiring signal for a specific nuclei, even ignoring physiological concentration differences. This value is typically normalized to that of the ^1^H nucleus to allow easy comparisons between the various nuclei. Receptivity values for various X-nuclei are shown in Table I. However, the receptivity still assumes equal concentrations of the various nuclei, and the fact that *in vivo* concentrations for X-nuclei are typically significantly lower than that of ^1^H can make these nuclei even harder to detect. For instance, even ^23^Na which has a comparatively high receptivity compared to many X-nuclei still typically obtains a decreased SNR by a factor of 6000 compared to conventional ^1^H cardiac imaging [11].

**TABLE I:** Receptivity of Various X-Nuclei

Nucleus	Spin: $I$	Gyr. Ratio: $\gamma $ (MHz/T)	Abundance: ${A_{nat}}$ (%)	Receptivity: $R$
^1^H	1/2	42.57	99.99	1.00
^31^P	1/2	17.24	100.00	0.066
^13^C	1/2	10.71	1.11	0.00016
^23^Na	3/2	11.26	100.00	0.093
^19^F	1/2	40.06	100.00	0.83

Regardless of sensitivity challenges, X-nuclei are of interest to researchers in both spectroscopic and imaging applications, and the use of array coils is standard practice to address challenges in sensitivity. Before reviewing the past and current state of the use of array coils for X-nuclei work, a brief discussion of the broad categorizations of X-nuclei spectroscopy and imaging is given below.

### A. X-Nuclei Spectroscopy

Though currently not a part of standard clinical practice, MRS is an invaluable medical research tool allowing investigators to assess *in vivo* concentrations of metabolites within a subject to study disease progression, predict treatment response, and determine factors that might predispose certain populations to particular diseases. ^1^H, ^31^P, and ^13^C are among the most often studied nuclei in biomedical research applications of MRS with ^1^H being the most common [6], [12]–[15].

Despite sensitivities that are lower by orders of magnitudes, spectroscopy of X-nuclei can provide complementary information unavailable through ^1^H studies. This review focuses on hardware approaches to improve SNR. Though there are numerous sequence-based methods which can lead to substantially increased signal strength in spectroscopy experiments of X-nuclei such as proton decoupling [16] and the Nuclear Overhauser Effect (NOE) [17], MRS of X-nuclei is still fundamentally limited in its achievable SNR, making any hardware-based increases in SNR broadly applicable.

### B. X-Nuclei Imaging

X-nuclei imaging is also possible for nuclei with sufficient abundance. Here we are defining “imaging” as experiments in which intensity values rather than full spectra are obtained directly from each voxel. This definition differentiates imaging from multi-voxel spectroscopic techniques such as CSI (also referred to as MRSI) in which distinct spectra are obtained per voxel even if these datasets are later viewed as images by color-mapping specific metabolite peak intensities for each voxel. Presently non-^1^H imaging is limited to ^23^Na for chemicals occurring naturally within the body though other X-nuclei can be attractive for imaging when injected. Though inferior to ^1^H MRI in terms of achievable spatial resolutions and anatomical data, imaging studies of X-nuclei can still offer important information not available through ^1^H imaging and have been utilized for the study of numerous disease processes.

Going forward, techniques such as enrichment or hyperpolarization [18] are creating opportunities for MR spectroscopic imaging in nuclei including ^3^He, ^129^Xe, ^13^C, and ^15^N. In these cases, adequate image resolution and SNR can often be achieved using basic surface coils, but array coils can still be used to decrease scan time using accelerated imaging techniques.

The objective of this work is to review the state of RF array coil adoption for non-^1^H studies. The work begins by providing a brief overview of the theory and benefits of array coil usage in general. Separate sections are next included for the most commonly encountered X-nuclei including ^31^P, ^13^C, ^23^Na, and ^19^F, such that the biomedical applications and study difficulties specific to each nuclei may be discussed separately along with limitations of more traditional coil designs Reviews of past and current RF array coil usage for these nuclei are then given for each.

## II. RF Array Coils

The most common instrumentation approach used to increase SNR in ^1^H MR experiments is to improve the sensitivity of the radiofrequency (RF) coil(s) used during reception of the signal from the sample. As the first component within the signal reception chain, the RF coil determines the maximum achievable SNR of the magnetic resonance experiment. It is therefore imperative that the RF coil be optimized to achieve maximum SNR throughout the desired region of interest.

The most basic coil used for MR signal reception is a surface loop coil which was first demonstrated by Ackerman et. al for spectroscopic studies [19] and later adopted by Axel for imaging applications [20]. This coil type is sensitive to signal loosely contained in a hemispherical region adjacent to the coil. Smaller loop coils offer superior sensitivity near the coil due to closer proximity of coil conductors to the region of interest and a decreased “noise volume”, the region from which noise is received. However, the sensitivity of these coils falls off rapidly as the distance from the coil increases, with the “sensitive region” of a surface loop generally considered to be approximately equal to its diameter. Therefore, when using a single loop coil, a trade-off must be made between shallow-depth sensitivity of the coil and coil sensitivity deeper within the sample.

**Fig. 1.**
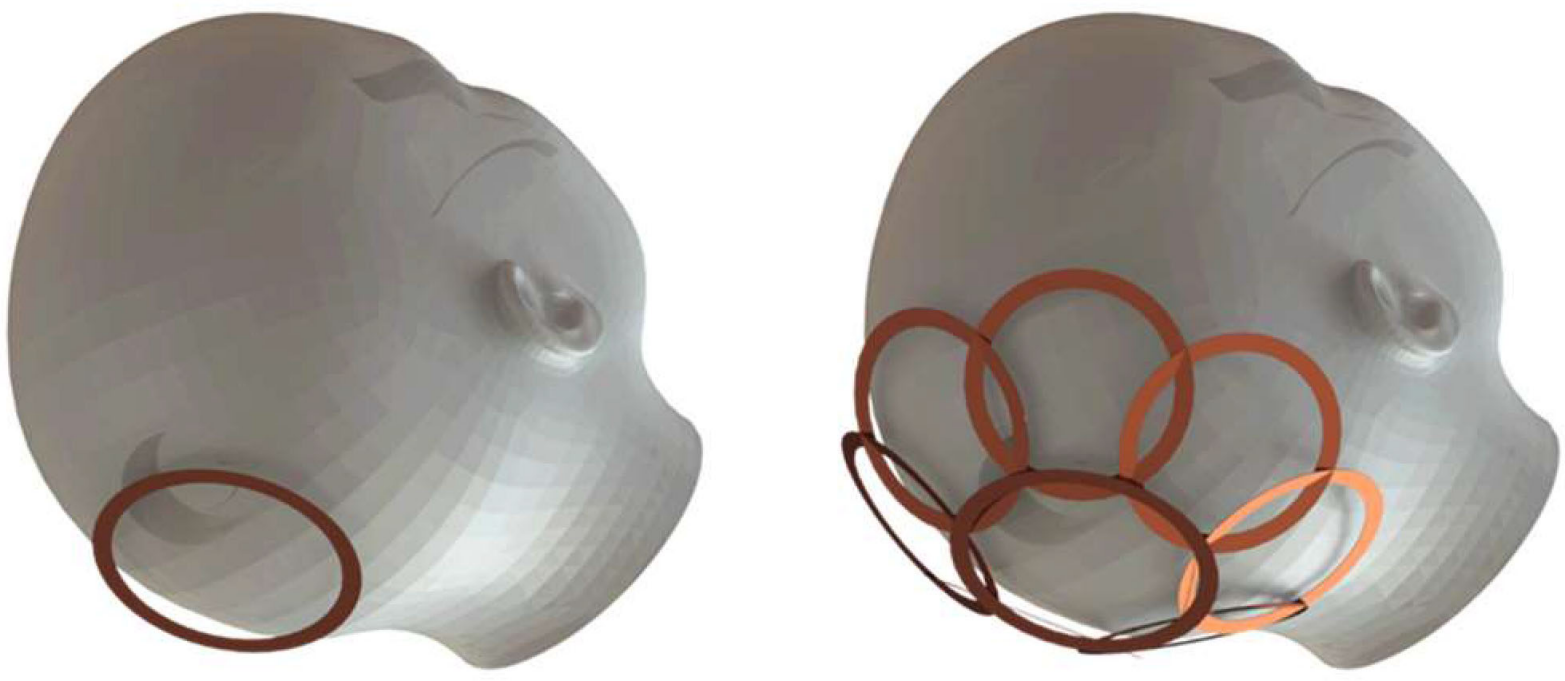
Renderings of typical single-element (left) and array coil (right) setups. RF array coils are commonly comprised of multiple (often but necessarily overlapped) loop elements conformed to the anatomy of interest to retain the benefits of small loop coils over an extended field-of-view.

**Fig. 2.**
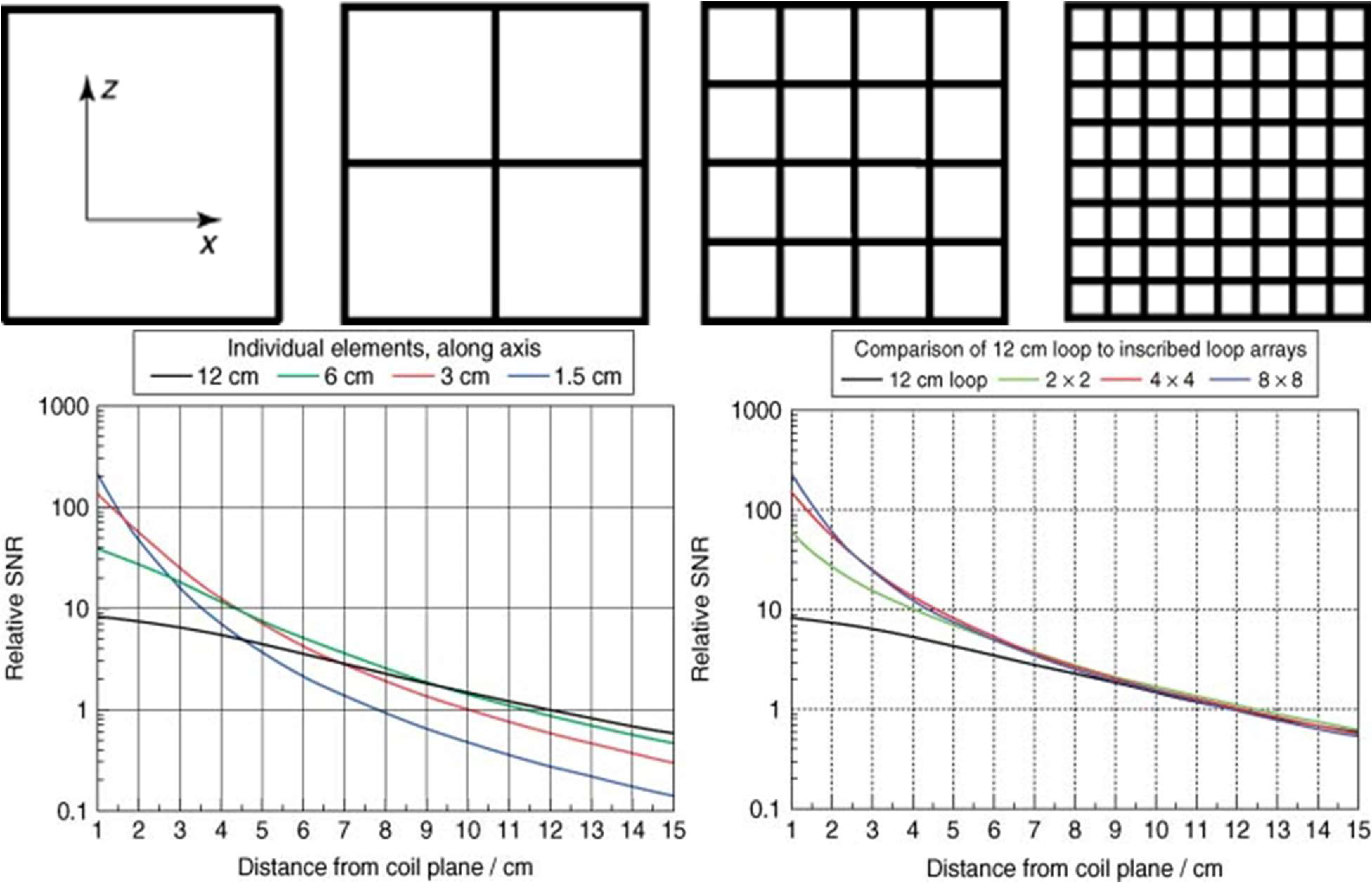
Calculated SNR comparisons of square, single-loop elements of varying sizes (bottom left) to array coils with overall dimensions of 12x12 cm but made up of increasing smaller elements (bottom right). Coil subdivisions for the various configurations are shown above the plots. When used as single elements, the relative SNR is highest near the coil surface for the smallest elements but quickly falls off compared to larger elements as imaging depth increases. However, with the elements combined in an array configuration, the array using the smallest elements retains high surface SNR while simultaneously maintaining the relative SNR of larger elements at increased imaging depths [25]. In contrast, a volume coil encompassing the entire imaging region and with similar height and width dimensions to the large loop coil would be expected to have a relatively flat SNR profile within the entire region but with decreased surface SNR. Reprinted by permission from Copyright Clearance Center: John Wiley and Sons EMAGRES Receiver loop arrays, Steven M. Wright, 2011.

In order to obtain the high SNR of smaller loop coils over an extended field-of-view or at greater depths, array coils consisting of a number of small, independent coils are often utilized [4], [21]–[24] with individual coil elements most often arranged in cylindrical, hemispherical or planar configurations to closely conform to the anatomy of interest as seen in Figure 1. As demonstrated in Figure 2, signals from individual coils are combined to achieve the surface sensitivity of small loop coils while retaining the depth sensitivity and extended field-of-view of a larger coil [4], [24] or to perform image acceleration.

The most common goal of array signal recombination methods is to maximize SNR of each pixel or spectra. As described by Roemer, this almost invariably involves multiplication of the signals obtained from individual coil elements by a set of complex weighting coefficients prior to signal recombination [4]. Though exact calculation of weighting factors varies with different techniques, they are typically chosen such that the signals from array elements most sensitive to the region-of-interest contribute more substantially to the final image/spectra relative to less sensitive elements. Thus, signal is maximized while noise contributions are reduced. Calculation of the weighting coefficients can be accomplished using data obtained prior to scanning (i.e., coil field maps, noise correlation matrices, etc.) or more commonly inferred from the images themselves as in the case of the popular “sum-of-squares” recombination method [25].

For spectroscopy studies, the general goals and strategies of signal recombination are similar [22] and often utilize a single high-intensity peak such as the water peak as a reference signal [26]. Weighting factors based on the signal [27], SNR [28], or S/N^2^ [29] have been proposed with no clear consensus on which is optimal. Algorithms such as singular value decomposition [30]–[32], principal component analysis [33], and generalized least squares [34] along with numerous variations of each have also been explored and have led to improved results in some cases.

Array coils are now commonplace in both ^1^H imaging and spectroscopy and have also led to substantial benefits in imaging speed along with SNR gains. Researchers quickly recognized that array coils could be used to accelerate imaging [35]–[37]. Now termed parallel imaging, these techniques rely on using the distinct reception profiles of the individual receive elements to reduce the required density of k-space data acquired and lead to substantially reduced scan times. Among the most popular implementations of parallel imaging are SENSE [38] and GRAPPA [39] which are commonly available on commercial systems as well as SMASH [40], [41], a similar but less commercially common technique. Combined with the SNR improvements, this benefit has led to increasingly higher channel counts for clinical ^1^H coils, with 32 becoming commonplace and channel counts as high as 128 or higher now provided by the manufacturers in some cases, often in research settings [42].

For X-nucleus studies, array coils are not as prevalent mainly due to lack of available support hardware. Historically, most clinical scanners have offered only a single broadband X-nuclei receiver channel, limiting the use of array coils for these applications. Workaround techniques, such as frequency translation [43], [44] or multiplexing [45]–[47], have been explored to provide multi-channel capability on systems without multiple broadband receivers. Additionally, clinical scanners which offer multiple broadband receiver channels are slowly becoming more prevalent as the utility of multinuclear studies becomes more apparent.

Considering achievable SNR in most X-nucleus studies is often several orders of magnitude less than those in ^1^H studies, increasing SNR in these studies is of significant benefit, and the use of array coils can aid in this regard. Though challenging, current research is showing great success using these methods, and interest in the use of X-nucleus array coils is increasing.

Even when using an X-nucleus array coil, ^1^H imaging capabilities are still usually needed, so RF coil systems are often designed to obtain signal from both the ^1^H nucleus and X-nucleus. Though this is especially necessary in high-field (i.e., 7 T and above) scanners which are particularly suitable for studies of X-nuclei but do not typically contain a ^1^H body coil [48], additional ^1^H capabilities are desirable even at lower fields to obtain high-quality anatomical images. These images can then be co-registered with the lower resolution metabolic information provided by the X-nucleus. Additionally, including a ^1^H channel allows for shimming of the magnetic field which would be not practical with the X-nucleus channel. Finally, the ^1^H channel can often be useful for signal enhancement through proton decoupling [16] or the Nuclear Overhauser Effect [17]. Though basic array coil design principles used for ^1^H arrays apply equally well to X-nucleus arrays, the necessity of including both X-nucleus and ^1^H capabilities further complicates design of these arrays and makes them distinct from typical ^1^H arrays.

In cases where ^1^H sensitivity is not critical, large volume coils are often sufficient for both transmission and reception of the ^1^H RF field while a separate, nested receive coil array is used to maximize SNR of the X-nucleus. However, even in this case care must be taken to adequately decouple the X-nucleus coil from ^1^H circuitry. One popular strategy to prevent interactions between the two frequencies is to insert resonant trap circuits tuned to the ^1^H frequency into the elements of the X-nucleus coil [49]–[51] to minimize current flow on the X-nucleus element at the ^1^H frequency. This decreases coil coupling and minimize coil losses.

If array coil operation at both frequencies is desired, geometric decoupling [4], [21], [22], [52] can be used to minimize mutual inductance between array elements. This strategy is especially convenient for high-field designs utilizing the complimentary field patterns of dipole and loop elements to innately decouple the two element types [53], [54] and has been used for both increasing ^1^H sensitivity [55]–[58] and as a method of forming double-tuned arrays [59], [60].

An additional option is to double-tune each array element [61] using strategies such as the Schnall trap design [62], transformer-coupled design [63], [64], or Hyde loop gap resonator [65], [66] depending on the specific application. Though technically challenging, this method is especially advantageous because it ensures array operation with equal field-of-view at both frequencies. If truly simultaneous operation at both frequencies is not necessary, PIN diodes [67]–[70], varactor diodes [71], [72], or MEMS switches [73], [74] may instead be used to add or remove tuning elements into the element coil circuitry to achieve switch-tunable designs.

The benefits of the discussed tuning strategies along with others can be found in the work presented by Choi [75], but the specific choice of strategy for X-nuclei arrays is largely dependent on the specific application,. The next sections more fully explore past and current applications of these array coil designs for specific nuclei (^31^P, ^13^C, ^23^Na, and ^19^F) while providing an overview of some of the unique information available from each of these nuclei.

## III. ^31^P NMR

^31^P spectra offer a wealth of information not attainable through ^1^H spectroscopy, particularly for studying muscle energetics and the disease processes which affect them [76]–[80]. Additionally, ^31^P MRS returns relatively simple spectra and a large chemical shift range which ensures that separate metabolite peaks are easily distinguishable compared to those encountered in ^1^H spectroscopy [78]. Despite this decreased complexity, the spectra are still rich in information. Ratios of inorganic phosphate and phosphocreatine are easily measured and often used as an indicator of muscle activation, and pH can also be easily inferred from the ^31^P spectra [77], [78].

Muscle energetics have been studied using ^31^P in a number of muscle groups including calf [81], [82] and forearm [83] muscles with recent interest in studying the myocardium of the heart [27], [84]. ^31^P MRS is also useful in the study of cancer. Due to increased cellular proliferation, increases in phosphomonoester and phosphodiester peaks and decreased phosphocreatine levels are often observed in malignant tumors [76], [80], [85].

Despite its proven utility, adoption of ^31^P MRS for *in vivo* studies has been difficult. Inherently low sensitivity of the ^31^P experiments necessitates the use of small surface coils to obtain adequate SNR when spectra from smaller regions are desired. Until recently, this has limited most muscle studies to superficial muscles within the coil's small sensitive region. Even in studies of superficial muscles, coil positioning can be problematic. In fact, coil repositioning was and still is noted as one of the most significant factors in work flow in these studies [27].

The high heterogeneity of muscle tissue and different fiber types often demand that ^31^P spectra be localized to be most useful for muscle studies [81], [86], [87] as well as tumor studies where voxels completely localized to tumors are preferable [80]. Unfortunately, any localization method inherently results in lower sensitivity compared to non-localized studies. Historically, single voxel spectroscopic methods have been used to achieve localization. In his 1992 review of the field of skeletal muscle energetics, Chance notes the crude voxel sizes on the order of 25 cm^3^ which were necessary due to insufficient SNR of smaller voxels [79].

Largely facilitated by increases in magnetic field strengths and array coil technology, there have been tremendous advances in spectroscopic imaging methods which helps alleviate issues with coil positioning. ^31^P CSI voxel sizes have significantly decreased voxel sizes in these studies with Chen *et al.* reporting using approximately 7.5 cm^3^ voxels in the brain at 7 T in 2002 [88]. More recently, groups have reported voxels as small as 4.1 cm^3^ [89] and 3.4 cm^3^ [60]. However, there is still much room for improvement. Along with decreasing voxel sizes, SNR increases afforded by array coils can be traded for faster scan times so that dynamic studies can be performed, which is most often done for enrichment or hyperpolarization studies.

**Fig. 3.**
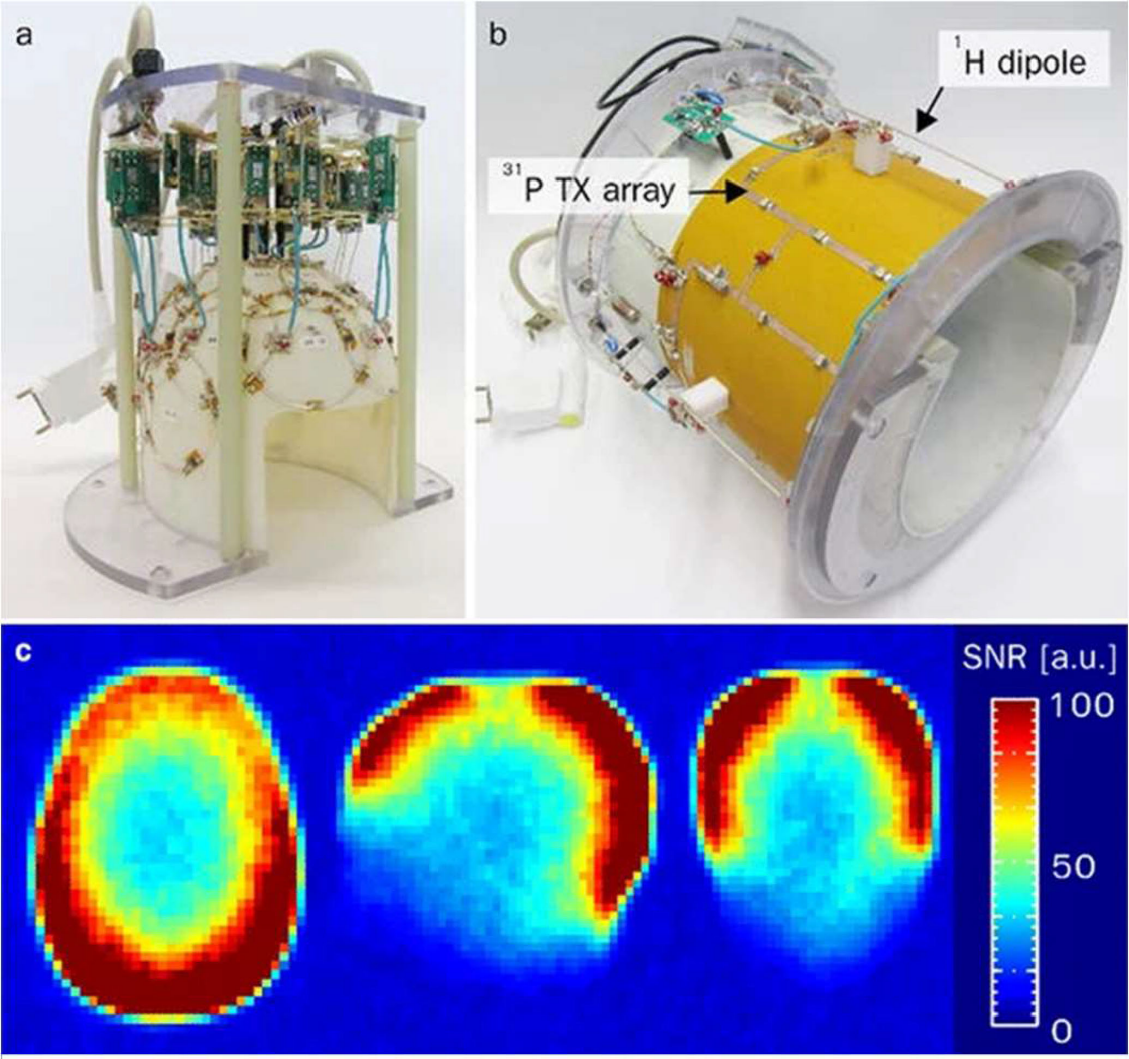
The multi-layer coil array presented by Mirkes et. al consisted of a 27-element receive-only ^31^P array (a) combined with 4-element ^31^P and ^1^H transceive arrays (b). As expected, the array displayed significantly increased peripheral SNR (c) and was used to obtain high-quality ^31^P CSI datasets with nominal 15x15x15 mm^3^ resolution within 22 minutes [65]. Compared to previous studies, the 9.4T field strength and close-fitting design enabled increased SNR even in the central brain regions. Reprinted by permission from Copyright Clearance Center: Springer Nature MAGNETIC RESONANCE MATERIALS IN PHYSICS, BIOLOGY AND MEDICINE 31P CSI of the human brain in healthy subjects and tumor patients at 9.4 T with a three-layered multi-nuclear coil: initial results, Christian Mirkes et al, 2016.

The idea of using array coils in ^31^P MRS was first demonstrated by Hardy *et al.* in 1992 with a three-channel, form-fitted coil that allowed a 2.5-fold increase in lateral field-of-view, increased imaging depth, and increased SNR within the anterior myocardium [27]. More recently, three-channel [90], eight-channel [85], sixteen-channel [84], and twenty-seven-channel [60] coils were used to study the calf, liver, heart, and brain respectively. All coils provided impressive SNR gains with Valkovic's *et al.* sixteen-channel array able to obtain phosphocreatine maps of the myocardium and increase SNR by a factor of 2.6 within the myocardium [84]. Likewise, Mirkes’ brain coil shown in Figure 3 demonstrated the feasibility of 3D CSI imaging of the brain within reasonable scan times [60].

**Fig. 4.**
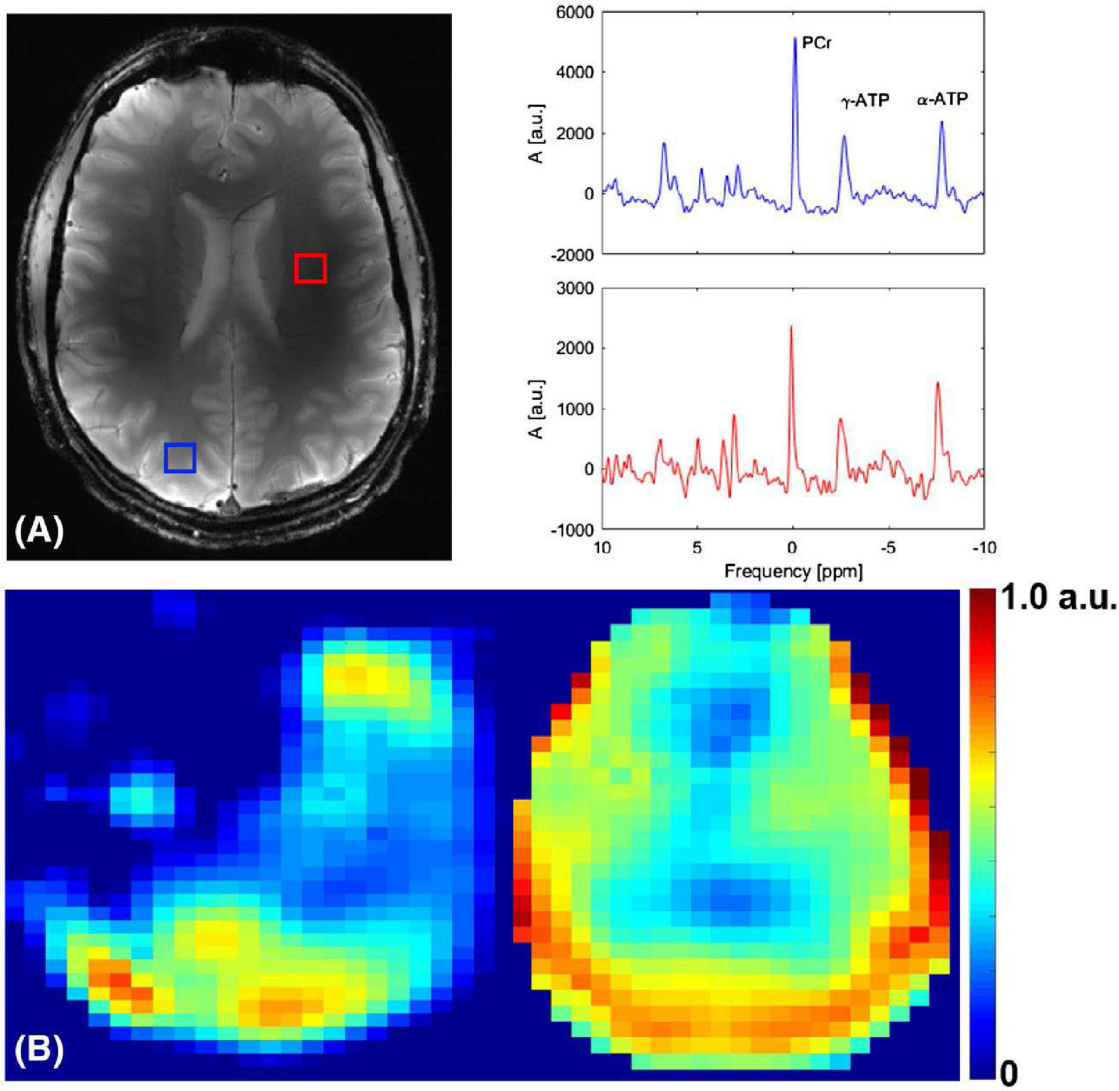
The 10-element ^31^P array coil array presented by Avdievich et. al maintained good central-brain sensitivity as demonstrated in the PCr maps (bottom row) with nominal 15x15x20 mm^3^ resolution. High-quality ^1^H images could be obtained (top left) with sample spectra (top right) shown indicated by blue and red boxes. Despite the design minimizing sensitivity inhomogeneities, the ^31^P spectra still show increased SNR in the periphery compared to the central brain as is typical of array designs. Reprinted with permission from Wiley Periodical, Inc.MAGNETIC RESONANCE IN MEDICINE Double-tuned 31P/1H human head array with high performance at both frequencies for spectroscopic imaging at 9.4T, Nidolai I. Avdievich and Loreen Ruhm, 2019.

Even more recently, Avdievich et. al reported a 10-element design intended to provide high SNR within the brain at 9.4T while maintaining good ^1^H coil performance and minimizing inhomogeneous sensitivity patterns of the coil [91] as shown in Figure 4. Gosselink et. al have also reported an 8-element ^31^P array which they used for signal reception at 7T while transmitting with an integrated ^31^P body coil. In this way, the array was able to provide optimal sensitivity while maintaining homogeneous ^31^P excitation. Finally, Rowland et. al have demonstrated in a 30-element multi-tuned ^31^P/^1^H array [61]. This array was particularly impressive considering each individual element was double-tuned which allowed for substantial increases in peripheral ^31^P SNR while maintaining good ^1^H imaging performance. Despite these impressive strides, the practice of using ^31^P array coils has yet to be widely adopted and these cases are still a relative rarity.

## IV. ^13^C NMR

^13^C is the second most commonly-used X-nuclei used in MRS. However, the low gyromagnetic ratio and the fact that only 1% of carbon within the body is the NMR-active ^13^C isotope [92] make it challenging to obtain adequate SNR to perform meaningful ^13^C studies using conventional techniques. Despite the difficulties, researchers are interested in ^13^C MRS studies due to its ability to examine numerous pathologies and metabolic pathways within the body. These studies are enabled by both the notable increase in chemical shift range of ^13^C compared to ^1^H spectra and the ubiquity of carbon-containing metabolites within the body. Because of the low natural abundance of ^13^C, hyperpolarized or ^13^C-enriched substances can be injected into the body, enabling specific metabolic pathways to be studied with reduced interference from background signal.

Utilization of ^13^C MRS has therefore increased recently due to advances in hyperpolarization techniques such as Dynamic Nuclear Polarization (DNP) which can increase ^13^C signal levels by factors of 10^3^-10^4^ or higher [18]. However, due to T_1_ decay effects, the enhanced signal is present a very limited time, and scans must be completed within minutes to account for these effects. Additionally, hyperpolarization studies are complicated by other issues such as RF saturation, substrate washout, and substrate metabolism [93]. In this case, with the high SNR available due to the hyperpolarization, array coil reception can dramatically reduce scan times by enabling parallel imaging techniques [38]–[40].

Hyperpolarized studies have been of interest in the study of brain metabolism [94]–[96], and hyperpolarized ^13^C CSI could also become an important tool for monitoring response to cancer therapies. These studies benefit from small voxel sizes to localize the signal, further increasing SNR demands and making array coil usage highly beneficial. Despite this, most studies to date have utilized only simple surface coils.

Array work for ^13^C is still in its infancy, but a few research groups have already shown promising results for hyperpolarized applications. In one study, Ohliger used an 8-channel array coil and advanced reconstruction techniques to obtain a 30x10x16 matrix of CSI data with isotropic 8 mm^3^ resolution and a total scan time of 13.4 s [93]. Tropp has reported a 3-element array used to study brain metabolism with similarly significant results [97]. A 31-channel array has also been reported by Mareyam et. al for imaging the brain at 3T [98]. Combined with a detunable volume transmit coil, they were able to acquire 20x20 dynamic 2D CSI datasets with 2 cm isotropic resolution and temporal resolution of three seconds. Of note, an 8-channel ^13^C breast array for 3 T is available through RAPID Biomedical and has recently been used for hyperpolarized studies by Gallagher et. al [99] which showed the feasibility of tracking pyruvate metabolism in breast cancer patients by observing the exchange of hyperpolarized ^13^C labels between the injected pyruvate and endogenous tumor lactate pools.

In ^13^C spectroscopy studies using naturally-abundant rather than hyper-polarized ^13^C, the need for increased SNR is more fundamental. Typically, small surface coils and long scan times must be used for these studies to distinguish different metabolite peaks due to the extremely low SNR. Using single coils, only organs close to the surface, such as the liver and superficial muscles, have been studied.

MRS studies of naturally abundant ^13^C in the liver have proven to be a viable tool for glycogen content measurement in studies of diabetic patients [100], [101] as well as the for investigating the role of diet therapy in disease treatment [102]. The SNR increase provided by array coils could provide an ability to discern less sensitive metabolites in deeper tissues in much the same way that these coils are beginning to be used for ^31^P studies, but thus far work in using array coils to detect naturally abundant ^13^C is in only preliminary stages [51].

## V. ^23^Na NMR

Of the X-nuclei discussed thus far, ^23^Na is unique in that it has no natural chemical shift making spectroscopic studies of ^23^Na less appealing than with other nuclei. However, the nucleus's 100% natural abundance allows suitable sensitivity for imaging experiments, and it is the most-commonly imaged X-nucleus (i.e., through the use of a frequency-encoding gradient during acquisition) in its naturally abundant state, though other nuclei such as ^17^O, ^39^K, and ^2^H are occasionally imaged. The ^23^Na nucleus was first imaged *in vivo* in 1983 when Hilal showed that infarcts caused by stroke could be clearly visualized by ^23^Na imaging [103]. Along with continuing investigations into its use in detecting and visualizing stroke, researchers have also shown the value of ^23^Na MRI for its role in visualizing tumors [104] as well as issues of the heart, kidney, and prostate [105]. In addition, ^23^Na imaging has been successful in evaluation of osteoarthritis even before morphological changes appear [106]–[110]. Many of these applications, as well as their associated challenges, are discussed thoroughly in Thulborn's review of the field [111].

One of the ultimate goals of ^23^Na imaging studies is to obtain completely quantitative measurements which relate the tissue sodium concentration to the images obtained. This goal has been generally difficult due to confounding factors such as the fact that the ^23^Na nucleus exhibits a spin state of 3/2 leading to rapid bi-exponential T_2_ decay effects [112], [113]. Dealing with this effect requires the use of ultra-short TE sequences, but progress has been made on this front [114]. In addition, sub-millimeter resolutions are often necessary in these studies to mitigate partial volume effects, especially in thin tissues such as cartilage [115], and these resolutions are not typically achievable with the SNR provided by standard RF coils.

In addition, the inherent sensitivity is still extremely low when compared to the ^1^H nucleus. Specifically, the lower gyromagnetic ratio leads to a sensitivity decrease of roughly 20 times as compared to ^1^H, and low sodium tissue concentrations further decrease the achievable signal levels. In total, the achievable ^23^Na signal is roughly 4,000 times weaker than that of ^1^H throughout most of the body [103], [112], which makes achieving sufficiently high-resolution images within clinically-feasible scan times using typical coil setups very difficult. Consequentially, improvements in ^23^Na imaging have always been connected with SNR increases through the availability of increased magnetic field strengths. However, use of RF receiver arrays offers a complimentary method of SNR improvement.

Volume coils providing homogenous sensitivity have been particularly popular in the past for quantitative ^23^Na imaging to avoid issues with the inhomogeneous coil profiles of surface coil arrays which can lead to quantification difficulties unless transmit and receive sensitivities are carefully measured and compensated for [112]. With the implementation of advanced parallel imaging reconstruction, these difficulties have been further mitigated, and quantitative ^23^Na imaging has now been demonstrated in the brain [116]. This resolves one problem with using array coils, further motivating their expanded use in spectroscopic studies.

**Fig. 5.**
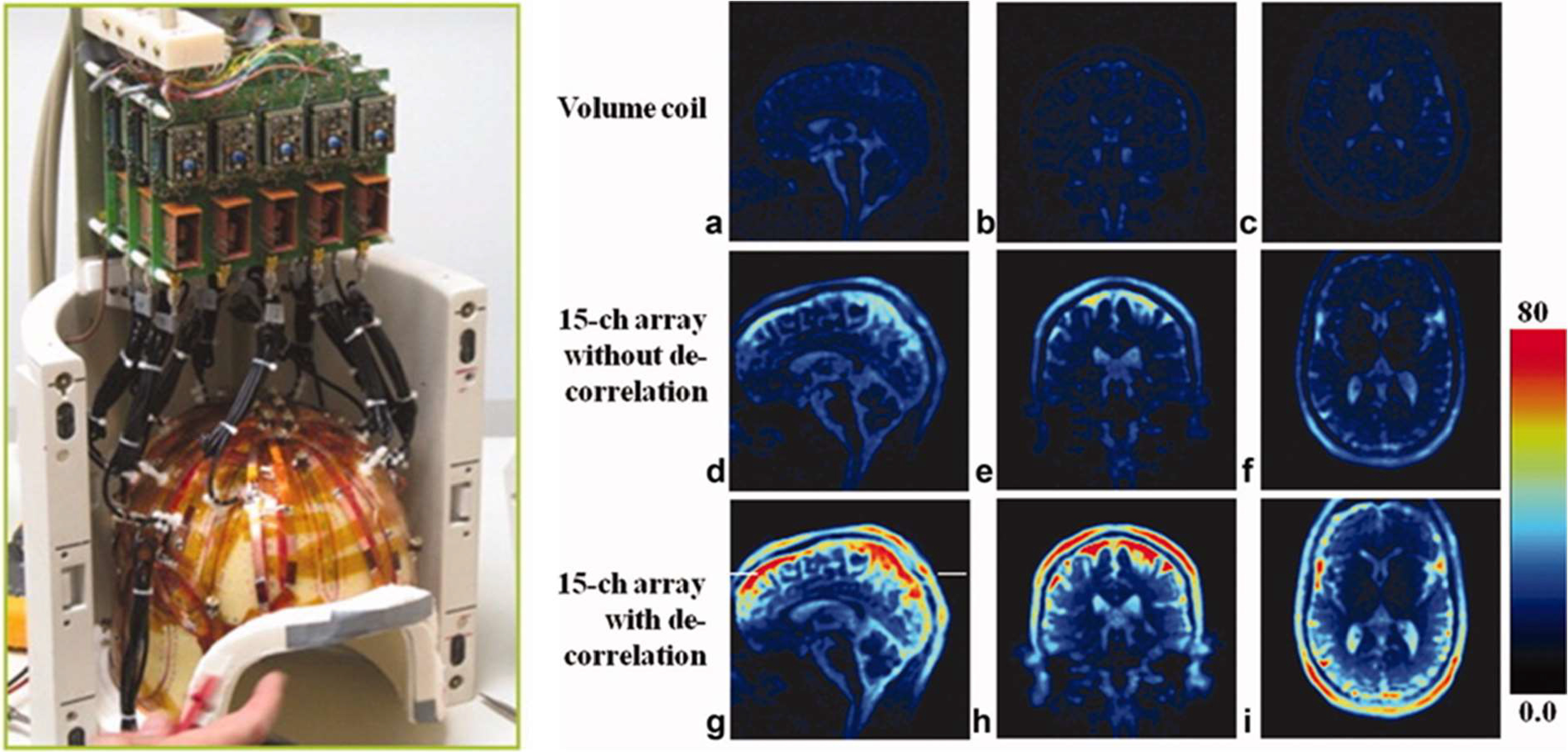
The 15-channel ^23^Na head array reported by Qian *et al* (left) showed increased SNR during brain imaging compared to a similar-sized birdcage volume transceive coil (a–c). In general, SNR increases were greater than a factor of two within the brain periphery with comparable central-brain SNR even when disregarding noise correlation between elements (d–f). De-correlation of noise further increased peripheral SNR and extended the 2x SNR region deeper into the central brain region (g–i) [96]. Reprinted by permission from Copyright Clearance Center: John Wiley and Sons MAGNETIC RESONANCE MATERIALS IN PHYSICS, BIOLOGY AND MEDICINE Sodium imaging of human brain at 7 T with 15-channel array coil, Fernando E. Boada, Jonathan Weimer, Hai Zheng, *et al*, 2012.

Of the X-nuclei, array coil usage has perhaps been most commonly implemented for ^23^Na studies [112]. The very first use of an array coil for ^23^Na imaging was by Bottomley *et al.* who used a frequency-translation approach with a 4-channel ^23^Na array [43] and reported 300-400% increases in SNR in certain voxels compared to a single-channel comparison coil. Since then, numerous other groups have begun seeing impressive results derived from the use of array coils. Array coils have been utilized by Wiggins *et al.* [117] as well as by Bae's group [115], [118] to obtain high resolution knee cartilage ^23^Na images with more than doubled SNR compared to volume coil designs. Another study in cadaver ankle joints achieved 1.79 mm^2^ resolution and quantitative *in vivo* sodium measurements using a 15-channel ^23^Na array [109]. Array coils for ^23^Na have also been developed by Ha *et al.* [68] and Bangerter *et al.* who used a 7-channel ^23^Na array to obtain SNR increases of 2-5 times within the breast at 3 T [113]. Qian et. al reported a 15-channel coil used to image the brain at 7 T in 2012 [119] which is shown in Figure 5. The array coil doubled the achievable SNR in the brain with even greater increases seen in the brain periphery compared to a volume birdcage coil. Similarly, Shajan *et al.* recently reported a 27-channel receive array which provided more than a five times improvement in SNR along the periphery of the head compared to a similarly-sized birdcage coil at 9.4 T [120].

Groups have also recently looked into 4-channel [68] and 8-channel [69] switchable array designs using PIN diode control to easily transition between imaging either ^23^Na or ^1^H. Because these designs do not require trap circuits for decoupling or tuning, they were simple to implement though at the expense of not being able to operate at both frequencies truly simultaneously. Similarly, a design implemented by Yan et. al combining a monopole ^1^H element and ^23^Na loop element forgoes the use of trap circuits, instead relying on the intrinsic geometric decoupling between elements [121]. This design has been shown to reduce losses on the ^23^Na channel at 7T and has been implemented in an 8-element array with concurrent RF excitation for both frequencies of each element through a single port [122].

Perhaps most notably, the highest channel-count ^23^Na array to date is a 30-channel, conformal head coil [123] developed by RAPID Biomedical which is now commercially available. As expected, the coil displays large SNR improvements (>2 times) near the periphery and similar SNR at the central region of the brain compared to a 30 cm diameter dual-tuned ^1^H/^31^P reference birdcage coil which the array is designed to fit snugly inside. The commercial availability of such a coil is an exciting step towards further adoption of ^23^Na array coils.

## VI. ^19^F NMR

^19^F is another very attractive nuclei for imaging or spectroscopic studies. The human body has no detectable background ^19^F signal [124], [125] making ^19^F MRI/MRS using injected fluorinated compounds a very useful technique since these studies can be performed without the need for background signal suppression. In most cases, perfluorocarbons (PFCs) are used as tags in these studies for applications such as monitoring drug pharmacokinetics, assessing tumor hypoxia, monitoring angiogenesis, or tracking stem cells within the body [124]–[126].

Feasibility of ^19^F MRI was demonstrated only four years after the implementation of ^1^H MRI but interest was limited due to technical concerns until relatively recently [124]. Nevertheless, ^19^F offers some notable advantages for imaging. Its gyromagnetic ratio is 94% of the ^1^H gyromagnetic ratio, it possesses a nuclear spin of ½, and its natural abundance is 100%. These factors lead to an overall sensitivity of 83% relative to the ^1^H sensitivity [125], [127] assuming equal numbers of spins. When used for spectroscopic studies rather than imaging, ^19^F offers the advantage of a wide chemical shift range over 200 ppm, making ^19^F spectra less susceptible to peak overlaps compared to more crowded ^1^H spectra [126].

Despite relatively high sensitivity, ^19^F still suffers major limitations in terms of signal strength. Detectable ^19^F signal in most studies is dependent on the total number of fluorine tags on injected PFCs as well as the quantity of appropriate receptors within the body to bind the PFCs [128]. Because doses of PFCs are limited in human studies [125], ^19^F signal strength is inherently limited and more sensitive RF coils must be used to improve SNR. Additionally, higher spatial resolutions are desirable in ^19^F MRI and MRS studies to avoid partial volume effects [126], [129], further necessitating improved RF coil sensitivity.

Designing coils for ^19^F/^1^H studies has traditionally been quite different than designing coils for other nuclei since the resonant frequencies of ^19^F and ^1^H only differ by 6%. Traditional methods of double-tuning through pole-insertion [62] are ineffective, but different techniques [130] such as the use of over-coupled resonator circuits [64], [128], [131] are now being used effectively with novel coil designs still being developed [67], [73]. Despite progress in single-channel coil design, adoption of ^19^F array coils has been minimal, and many designs are as-of-yet unproven in their application as array coil elements.

Regardless, there are some notable example of ^19^F array coils such as the design by Ji *et al.* who used an 8-element array to obtain ^1^H and ^19^F images and perform single-voxel spectroscopy on the human knee [126]. The array coil was able to obtain ^19^F images with 1.5 x 1.5 mm^2^ in-plane resolution in only three minutes. An eight-element array of dipole antennas with bandwidths large enough to cover both the ^19^F and ^1^H frequencies at 7 T was also used by Gorp et. al to maximize sensitivity and spatial coverage of ^19^F CSI of the liver [132] while simultaneously providing B_1_ shimming capabilities. Finally, a 6-element ^19^F coil capable of operating at both 1.5T and 3T field strengths has also been demonstrated which was also capable of imaging ^1^H at 1.5T and ^129^Xe at 3T [133]. The coil used a trapped design to achieve simultaneous tuning but reported minimal resistive losses. The success of the coils in these applications demonstrates the utility of arrays for ^19^F applications and should hopefully lead to further development of these coils in the future.

## VII. Conclusion

Despite proven advantages, usage of array coils in X-nuclei studies is still not standard though promising advancements are underway. Encouraging early results from research groups and recent advances in receiver availability are beginning to make these coils more popular. Further evidence of increasing popularity is the recent availability of commercial X-nucleus arrays, and it seems clear that development of these systems will continue. Though immediate future advancements will likely involve similar array designs applied across a range of different anatomies, other array developments perhaps exploring new multi-tuning strategies are also possible. For instance, arrays consisting of multi-tuned elements as opposed to nested or decoupled designs are still a rarity even among multinuclear arrays, and further exploration of this technology is likely.

Motivation for X-nucleus array coil development includes the desire to perform dynamic studies and/or provide better signal localization, particularly across a wider field-of-view. The trend of whole-body research scanners moving to higher field strengths is a driving factor for multinuclear studies and has brought forward the possibility of many relatively unexplored research areas which will further dictate the need for the most effective coil array designs. Recent studies combining the strengths of ultra-high fields and array coil technology for ^23^Na and ^31^P have already sparked substantial interest. However, these are not the only nuclei being explored, and in general we should expect to see continuing advances in the efforts to apply the benefits of RF array coils in the multinuclear space.
